# Network Pharmacology-Based Approach to Revealing Biological Mechanisms of Qingkailing Injection against IschemicStroke: Focusing on Blood-Brain Barrier

**DOI:** 10.1155/2020/2914579

**Published:** 2020-08-27

**Authors:** Shuang Zhang, Xueqian Wang, Fafeng Cheng, Chongyang Ma, Shuning Fan, Wenxiu Xu, Na Jin, Shuling Liu, Kai Lv, Qingguo Wang

**Affiliations:** ^1^Beijing Key Laboratory, School of Basic Medical Sciences, Beijing University of Chinese Medicine, 11 Beisanhuandong Road, Chaoyang District, Beijing 100029, China; ^2^School of Traditional Chinese Medicine, Capital Medical University, Beijing 100069, China; ^3^The Third Affiliated Hospital of Beijing University of Chinese Medicine, 51 An Wai Xiaoguan Street, Chaoyang District, Beijing 100029, China

## Abstract

Ischemic stroke is the most common type of cerebrovascular accident worldwide. It causes long-term disability and death. Qingkailing (QKL) injection is a traditional Chinese patent medicine which has been clinically applied in the treatment of ischemic stroke for nearly thirty years. In the present study, network pharmacology combined with experimentation was used to elucidate the mechanisms of QKL. ADME screening and target prediction identified 62 active compounds and 275 targets for QKL. Topological screening of the protein-protein interaction (PPI) network was used to build a core PPI network consisting of 408 nodes and 17,830 edges. KEGG enrichment indicated that the main signaling pathway implicated in ischemic stroke involved hypoxia-inducible factor-1 (HIF-1). Experimentation showed that QKL alleviated neurological deficits, brain infraction, blood-brain barrier (BBB) leakage, and tight junction degeneration in a mouse ischemic stroke model. Two-photon laser scanning microscopy was used to evaluate BBB permeability and cerebral microvessel structure in living mice. HIF-1*α*, matrix metalloproteinase-9 (MMP-9), and tight junction proteins such as occludin, zonula occludins-1 (ZO-1), claudin-5, and VE-Cadherin were measured by western blotting. QKL upregulated ZO-1 and downregulated HIF-1*α* and MMP-9. QKL has a multiapproach, multitarget, and synergistic effect against ischemic stroke.

## 1. Introduction

Stroke is a major cause of long-term disability and death worldwide. Despite the stable morbidity of stroke and the decline in its mortality over the past decade, the absolute numbers of incidences and disability-adjusted life-years associated with it are rising. Moreover, the global burden of stroke may increase as the population ages [1]. Ischemic stroke is the most common type of cerebrovascular accident. It accounts for approximately 80% of all types of strokes [2]. It is the result of the thromboembolic occlusion of a major cerebral artery or its branches [3]. Recombinant tissue plasminogen activator (rt-PA) is the most effective and the only approved therapy for ischemic stroke [4, 5]. Nevertheless, treatment of ischemic stroke with intravenous thrombolysis is limited by a short therapeutic time window, incomplete recanalization, hemorrhagic transformation, and reperfusion injury [3].

The blood-brain barrier (BBB) is a highly specific vascular interface maintaining brain homeostasis by separating the blood compartment from the central nervous system (CNS) [6]. Recent studies showed that ischemia/reperfusion injury is an important environmental stress factor in ischemic stroke. It is associated with disruption of the BBB [7, 8]. After reperfusion, the activated matrix metalloproteinase (MMP) system [9], damaged basement membrane [10], and overreactive inflammatory response [11] induce BBB damage. The latter has serious consequences such as cerebral edema, hemorrhagic transformation [12], and neuronal injury [13]. Evidence from in vivo and in vitro studies indicates that the activation of HIF-1 pathway is an important contributing factor in BBB damage after acute ischemia stroke [14, 15]. Therefore, the reduction of BBB permeability induced by HIF-1 activation is a necessary effect of future cerebral ischemia therapies.

Qingkailing (QKL) injection is a traditional Chinese patent medicine approved by the China Food and Drug Administration (CFDA, http://www.sda.gov.cn) for the clinical treatment of acute stages of ischemic stroke. QKL is composed of eight medicinal materials or extracts, namely, *Isatis tinctoria* L., *Lonicera confusa* DC., Concha Margaritifera Usta, *Betula pubescens* var. *pubescens*, *Gardenia jasminoides* J. Ellis, cholic acid, hyodeoxycholic acid, and Cornu Bubali [16]. Systemic reviews and meta-analyses published by our group and others indicated that QKL combined with conventional therapy was more efficacious than a control treatment [17, 18]. Traditionally, the study of Chinese medicine such as QKL is designed in the mode of “one-target, one-drug.” In recent years, however, researchers began to introduce the concept of “network-target, multiple-component therapeutics” into the pharmacological research of traditional Chinese medicine [19, 20]. Under the guidance of this concept, network pharmacology has been successfully used in several studies on Chinese medicine [21–23].

In the present study, a network pharmacology approach was used to elucidate the mode of action QKL in the treatment of ischemic stroke. Protein-protein interaction network topological analysis and KEGG signaling pathway enrichment disclosed that HIF-1 is an important signaling pathway participating in the anti-ischemic stroke effect of QKL. A transient middle cerebral arterial occlusion (tMCAO) mouse model was used to establish the protective effects of QKL against BBB leakage, cerebral infarction, and expression of HIF-1*α*, MMP-9, and tight junction proteins.

## 2. Materials and Methods

### 2.1. Reagents and Instruments

The QKL injection was composed of Radix Isatidis, Flos Lonicerae, Fructus Gardeniae, Cornu Bubali (powder), Concha Margaritifera Usta (powder), Baicalinum, Acidum Cholicum, and Acidum Hyodesoxycholicum and was purchased from the Shenwei Pharmaceutical Group Co. Ltd., Shanghai, China (no. 171223A2). Edaravone (Simcere Pharmaceutical Co., Ltd., Nanjing, China) is a free-radical scavenger developed as a neuroprotectant for ischemic brain stroke, and it provides benefits in the treatment of acute brain infarction in animals and humans [24, 25]; it was used as a positive control.

### 2.2. Data Mining

Data on the individual herbs, and the ingredients of in Qingkailing injection and its targets were mined from the TCM systems pharmacology (TCMSP) [26] and BATMAN-TCM [27] databases. Known ischemic stroke-related targets were identified from the following five existing resources: (1) the DrugBank database: interactions were identified among FDA-approved drugs for ischemic stroke treatment and human gene/protein targets [28]; (2) the OMIM database [29]; (3) the GAD database [30]; (4) the TTD database [31]; and (5) Disease Gene Search Engine with Evidence Sentences (DigSee) using “Ischemic stroke” as the keyword [32]. All gene names were extracted from the UniProt Knowledgebase.

### 2.3. Prediction of Active Compounds in QKL

TCM formulations are composed of multiple compounds. However, not all of them are pharmacologically active. We screened various compounds present in QKL according to their pharmacokinetic ADME parameters, such as BBB (blood-brain barrier) [33] and DL (structural similarity between the constituents and clinically used drugs in the DrugBank database) [34], according to previously reported models [35]. Active compounds were selected on the basis of the threshold values of BBB ≥ −0.3 and DL ≥ 0.18.

### 2.4. Drug-Target Network and Protein-Protein Interaction Network Construction

The drug-target network was visualized with Cytoscape v. 3.5.1. PPI networks were constructed using a Cytoscape plugin (BisoGenet) to analyze five existing PPI databases: the Biological General Repository for Interaction Datasets, Human Protein Reference Database, Molecular Interaction Database, Biomolecular Interaction Network Database, and Database of Interacting Proteins [36].

### 2.5. Identification of Candidate Targets of QKL Responsible for Its Anti-Ischemic Stroke Effects

We first constructed an interaction network for the known ischemic stroke-related targets and predicted putative pharmacological targets of QKL based on the data obtained from BisoGenet. Further, the interaction network was visualized with Cytoscape. The topological properties of each node in the interaction network were assessed with the Cytoscape plugin (CytoNCA) on the basis of DC, BC, CC, EC, NC, and LAC. The definitions and computational formulas of these parameters were previously described [37]. Their values are directly correlated with their importance in the network. Topology was screened twice, and all targets in the core PPI network were considered as candidate targets.

### 2.6. GO and KEGG Pathway Enrichment Analysis

A DAVID-based GO enrichment and KEGG enrichment were performed to identify the genes involved based on the terms (BP, CC, and MF) and the KEGG pathways. At *P* < 0.05, a hypergeometric test was run to identify enriched GO and KEGG terms. An overview is shown with 10 significantly enriched terms related to ischemic stroke in each GO and KEGG enrichment.

### 2.7. Animals

All the experiments involving animals and their care were approved by the Animal Care Committee of Beijing University of Chinese Medicine (BUCM-4 20171015074007) and conducted in accordance with institutional guidelines and NIH Guide for the Care and Use of Laboratory Animals, NIH publication no. 85–23, revised 1996. Two hundred adult male C57BL/6 mice (20–24 g) were purchased from Vital River Laboratories, Beijing, China (no. SCXK (Beijing) 2006-0009). The mice were housed in the Central Animal Laboratory of the Beijing University of Chinese Medicine with free access to food and water. To ensure animal welfare, some measures were taken, such as placing wet diet pellets directly onto the cage floor, intraperitoneal (i.p.) injecting of 200 *µ*L of sterile prewarmed 0.9% NaCl solution preoperatively, using bupivacaine (2 mg/kg sc.) as local analgesia to the incision site, and performing observations and completing welfare sheets every 6 hours.

### 2.8. Transient Middle Cerebral Artery Occlusion

The ischemic stroke rat model was induced by tMCAO as previously described [38]. Briefly, after anesthetizing the mice with sodium pentobarbital (60 mg·kg^−1^, i.p.), the right common carotid arteries (CCA) and ipsilateral external carotid arteries (ECA) were exposed. The ECA were then distally ligated, and the internal carotid arteries (ICA) were exposed and clamped with microvascular clips. A 5-0 surgical nylon monofilament was gently inserted through the ECA, the carotid bifurcation, and the ICA until it reached the origin of the middle cerebral artery (MCA). After occlusion for 1 h, reperfusion was initiated by withdrawing the monofilament. For sham animals, all the protocols were performed in a similar way to the establishment of MCAO model, except the occlusion of MCA. To ensure the survival rate after surgery, body temperature was maintained using an electric blanket.

The mice were randomly divided into three groups: sham (Sham), middle cerebral artery occlusion (MCAO) (Ischemia), and Qingkailing (QKL), with six animals per group. The final QKL dose injected was 9 mL·kg^−1^. The route of drug administration is intraperitoneal injection. The first injection was performed immediately after model establishment. Subsequent treatments were administered 4 h later and once every 12 h thereafter [39]. The 24 h time point was selected to elucidate the underlying mechanisms of the effects of QKL on BBB dysfunction in MCAO mice. The peri-infarct areas of the brain were analyzed to report the cerebrovascular changes.

### 2.9. Neurological Assessment

All mice underwent Bederson neurological tests [40] at 24 h after surgery as previously described. There were four functional levels: 0 points: there were no symptoms of nerve damage; 1 point: contralateral front paw could not be fully extended in the tail suspension experiment; 2 points: there is decrease in forelimb resistance to the opposite side in the thrust ability assessment; and 3 points: mice turned to the opposite side.

### 2.10. Brain Infarct Volume

Brain infarct volume was assessed with triphenyltetrazolium chloride (TTC) staining [41] at 24 h following reperfusion. Brains were sectioned coronally into 2 mm slices and incubated in 2% TTC in phosphate-buffered saline (PBS) at 37°C for 10 min. The slices were fixed in 4% paraformaldehyde (PFA) for 24 h and scanned. The ischemic area and total area of each section were measured by ImageJ (NIH, Bethesda, MD, USA). The infarct volume was calculated as follows: corrected infarct volume = [{contralateral hemisphere volume − (ipsilateral hemisphere volume − infarct volume)}/contralateral hemisphere volume] × 100%.

### 2.11. BBB Permeability

BBB integrity was assessed by measuring the leakage of Evans Blue (EB; Sigma-Aldrich Corp., St. Louis, MO, USA) as previously reported [41], with some modifications. Briefly, a 2% EB solution in physiological saline (4 mL·kg^−1^ body weight) was injected at 4 or 24 h following reperfusion. The route of EB injection is tail vein injection. After the dye circulated for 1 h, the brain tissues were homogenized in 50% trichloroacetic acid (TCA) solution. The supernatant was diluted with a 3x volume of 100% ethanol and analyzed in a spectrofluorometer (Thermo Fisher Scientific, Waltham, MA, USA) at an excitation wavelength of 620 nm and an emission wavelength of 680 nm. The EB concentration was normalized to tissue weight (ng·g^−1^).

### 2.12. Nissl Staining

After being anesthetized with sodium pentobarbital (60 mg·kg^−1^, i.p.), the mice suffered perfusion with normal saline via the heart, followed by 4% PFA perfusion. The brain tissue was then fixed in the fixative for 24 hours and embedded in paraffin wax. A series of 5 um thick slices were cut on a rotary slicer for Nissl staining. The intact cells in the ischemic cortical penumbra highlighted by Nissl staining were counted at five randomly selected sites of injury [42]. The following scores were used to evaluate necrotic neurons in the infarct area: 0, normal; 1, <25% damaged neurons; 2, 25–50% damaged neurons; 3, 50–75% damaged neurons; and 4, >75% damaged neurons [43].

### 2.13. Two-Photon Laser Scanning Microscopy (TPLSM) In Vivo

A midline incision was made on the scalp to expose the surface of the skull. A cranial window of 3 mm diameter was opened with a dental drill through a stereotactic device (craniotomy window center: 2.5 mm front, 2.5 mm right) on the right parietal cortex. A coverslip of 8.0 mm diameter was glued to the top of the cranial window.

The peri-infarcted zones in the tMCAO mice were observed by TPLSM (FVMPE-RS; Olympus Corp., Tokyo, Japan). To assess BBB integrity [44], 0.1 mL fluorescein isothiocyanate-labeled dextran (FITC-dextran, 10 kDa, 100 mg·mL^−1^; Sigma-Aldrich Corp., St. Louis, MO, USA) was injected through the tail vein at 4 h after tMCAO to visualize the degree of extravasation of the brain microvessels. Capillaries 50 *μ*m below the surface of the cerebral cortex of each mouse were randomly selected to observe changes in BBB permeability and morphological destruction of the brain microvessels.

### 2.14. Western Blotting

Proteins from the peri-infarcted cerebral cortex in the ipsilateral hemisphere at 24 h after MCAO were separated by electrophoresis on 10% polyacrylamide gels and transferred to nitrocellulose blotting membranes. The latter were blocked; incubated with primary antibodies against HIF-1*α*, MMP9, ZO-1, VE-Cadherin, claudin-5, and occludin (1 : 1000, 1 : 1000, 1 : 500, 1 : 500, 1 : 1000, and 1 : 1000, respectively; Abcam, Cambridge, UK) and a secondary antibody (1 : 5,000; Santa Cruz Biotechnology, Dallas, TX, USA); and scanned by Odyssey (LI-COR, Lincoln, NE, USA). The density of each band was semiquantified with image analysis software.

### 2.15. Statistical Analysis

Data were expressed as means ±SEM. Statistical analysis was performed by SPSS software (IBM Corp., Armonk, NY, USA). One-way ANOVA was used to determine the significant differences among multiple independent samples. Data failing to meet the homogeneity of variance criterion were subjected to nonparametric tests (Kruskal–Wallis followed by Mann–Whitney U). Differences were considered statistically significant at *P* < 0.05.

## 3. Results

### 3.1. QKL Possessed Neuroprotective Effect on Ischemic Stroke

**Figure 1:**
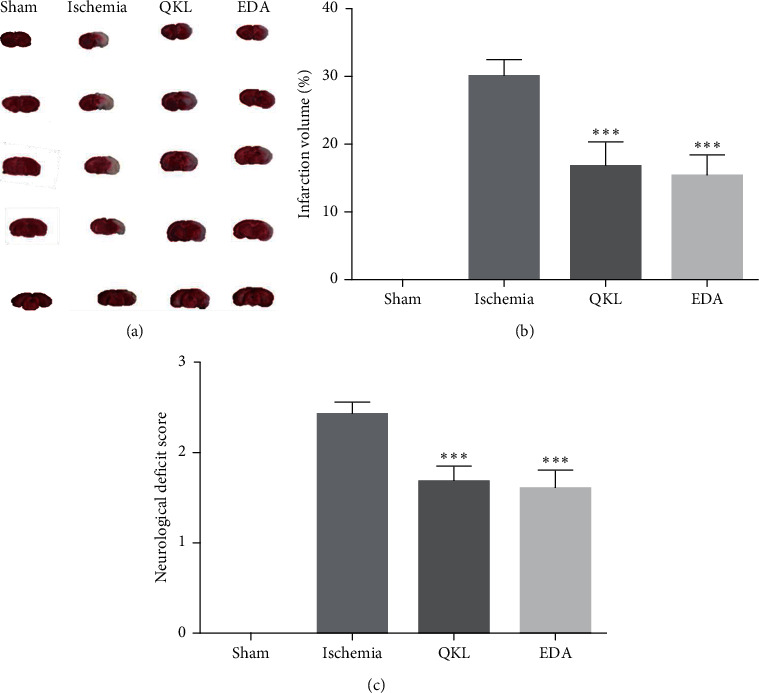
Infarction areas and neurological deficit score at 24 h after reperfusion. (a) Representative images of TTC-stained brain slices. (b) Quantitative analysis of cerebral infarct volume. (c) Effect of QKL on neurological deficit score. Data are reported as means ± SEM. ^*∗∗∗*^*P* < 0.001 vs. Ischemia group, ^*∗∗*^*P* < 0.01 vs. Ischemia group, ^##^*P* < 0.001 vs. Sham group (*n* = 6 per group).

To evaluate QKL anti-ischemic stroke efficiency, we used a tMCAO mouse model to mimic cerebral ischemia and reperfusion injury. The brain infarct volume was examined by TTC staining at 24 h. QKL significantly reduced brain infarct volume relative to the Ischemia group (Figures 1(a) and 1(b)). The neurological deficit score was also used to evaluate the antistroke effect of QKL. The mice in the QKL group had significantly lower neurological deficit scores than those in the Ischemia group (Figure 1(c)). Therefore, QKL improved neurological deficits and had a neuroprotective effect on ischemic stroke.

### 3.2. Screening of the Active Compounds in QKL and Constructing of Compound-Target Network

**Figure 2:**
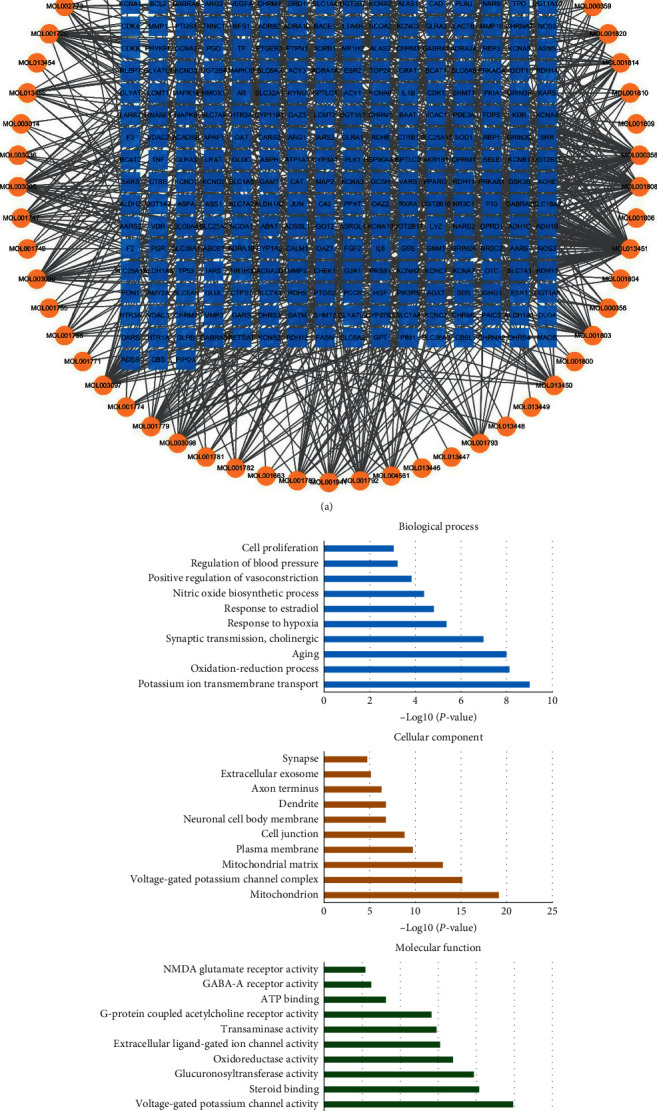
Screening of Bioactive compounds in QKL injection. (a) Compound-target network of QKL injection. (b) GO enrichment of QKL targets.

The active compounds in QKL had to be screened in order to elucidate its underlying mechanisms of ischemic stroke treatment. In the present study, the active compounds in QKL were screened for absorption, distribution, metabolism, excretion, and the ADME-related pharmacokinetic parameters BBB and DL. The screening criteria were BBB ≥ −0.3 and DL ≥ 0.18. After ADME screening and target searching, 62 active compounds and 275 targets of QKL were identified. A compound-target network consisting of 337 nodes and 994 edges was constructed (Figure 2(a)). According to the GO enrichment (Figure 2(b)), the QKL targets participated in oxidative stress, hypoxia, vasoconstriction, blood pressure, cell junction, and excitatory and inhibitory amino acid receptor, including NMDA glutamate and GABA-A receptor.

### 3.3. Intersection of the QKL and Ischemic Stroke-Related Targets

**Figure 3:**
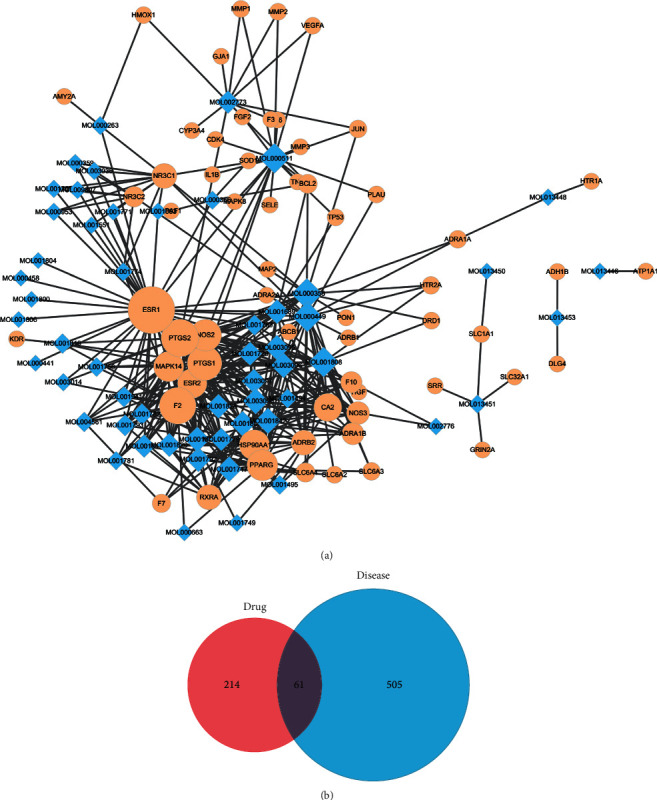
QKL targets related to ischemic stroke. (a) Compound-ischemic stroke-related targets network. (b) Venn plot of drug-targets and ischemic stroke targets.

A drug is administered according to its targets. Thus, we collected 566 ischemic stroke-related targets from the online DrugBank, OMIM, GAD, TTD, and DigSee databases. Sixty-one targets were identified, and a compound-ischemic stroke-related target network was constructed (Figures 3(a) and 3(b)). The ten leading compounds were *β*-sitosterol; stigmasterol; ursolic acid; tryptanthrin; pinoresinol; 3-hydroxy-2′,4′,7-trimethoxyflavone; erucic acid; tetracosane; sinensetin; and 5-hydroxy-3′,4′,5′,7-tetramethoxyflavone. Based on degree value, the top 10 targets were ESR1, PTGS2, F2, PTGS1, NOS2, PPARG, HSP90AA1, MAPK14, ESR2, and CA2.

### 3.4. Identification of the Candidate Targets and KEGG Pathways of QKL against Ischemic Stroke

**Figure 4:**
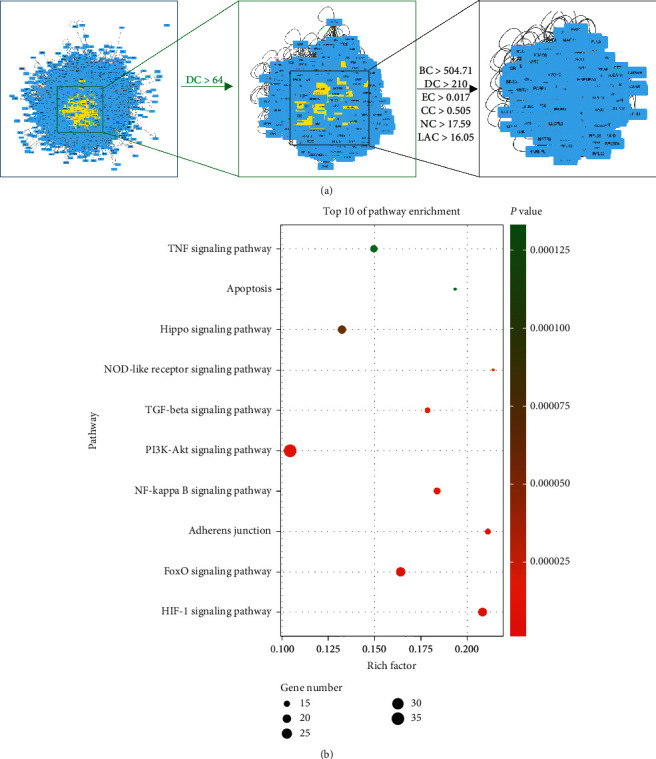
(a) Topological screening of PPI network. (b) KEGG enrichment of proteins in core PPI network.

Protein-protein interactions (PPIs) are important in many cellular responses. Modulation of the PPI network is a potential pharmacological mechanism [45]. To understand the anti-ischemic stroke effect in protein-protein interaction (PPI) network level, we constructed two PPI networks based on two different collections of seed proteins, QKL targets (5868 nodes and 144843 edges) and ischemic stroke targets (9778 nodes and 209705 edges), respectively. We intersected the two networks and obtained a new PPI network consisting of 5187 nodes and 137181 edges. The six topological features calculated by CytoNCA and used to identify candidate targets are “degree centrality (DC),” “betweenness centrality (BC),” “closeness centrality (CC),” “eigenvector centrality (EC),” “network centrality (NC),” and “local average connectivity (LAC).” We obtained a PPI network comprising 408 nodes and 17830 edges (Figure 4(a)). A KEGG enrichment analysis of the candidate targets in this PPI network disclosed 10 ischemic stroke-related signaling pathways, namely, HIF-1, FoxO, adherens junction, NF-kappa B, PI3K-Akt, TGF-beta, NOD-like receptor, Hippo, apoptosis, and TNF (ordered in *P* value, Figure 4(b)).

### 3.5. QKL Attenuated BBB Disruption Induced by Ischemic Stroke

**Figure 5:**
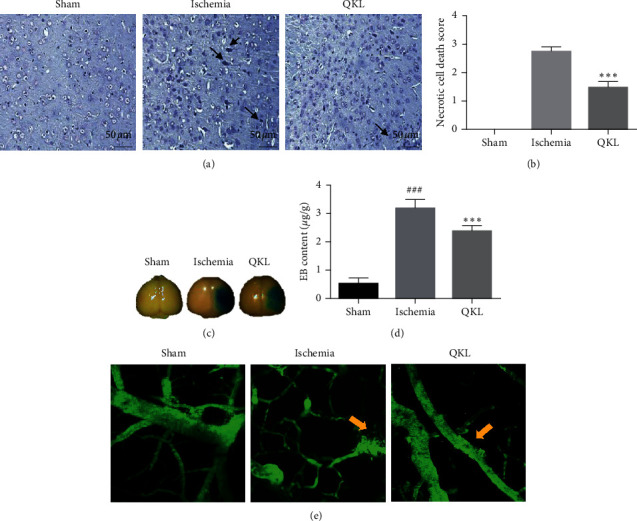
QKL possessed neuroprotective effect and attenuated BBB disruption. (a) Nissl-stained brain tissues after MCAO/reperfusion treatment (magnification ×400). (b) Bar graph indicates necrotic cell death score in penumbra area for each group. (c) Representative figure of BBB leakage evaluated by Evan Blue (EB). (d) Quantitative analysis of EB leakage. (e) FITC-dextran leakage in live mice. Data are reported as means ± SEM. ^###^*P* < 0.001 vs. Sham group, ^*∗∗∗*^*P* < 0.001 vs. Ischemia group (*n* = 6 per group).

Nissl staining showed that the brain tissue of the Sham group exhibited no significant pathological changes and its cell morphology was normal. In contrast, edema, necrosis, and shrink infarction were visible in the peri-infarct area of the cerebral cortices of the Ischemia group (Figure 5(a)). Semiquantitative morphological analysis showed that QKL lowered the necrotic cell death score relative to the Ischemia group (Figure 5(b)). A network pharmacological analysis of QKL indicated that 28 targets were expressed in the cell junction. The KEGG enrichment showed that the pharmacological effects of QKL against stroke involved the adherens junction pathway. As for the role of the cell junction in BBB structure and function, we assumed that QKL attenuated BBB disruption induced by ischemic stroke. Our quantitative results showed that EB in the Ischemia group was significantly higher than that in the Sham group. Therefore, BBB was disrupted after tMCAO. QKL treatment significantly diminished the EB leakage induced by brain ischemia/reperfusion (Figures 5(c) and 5(d)). We used two-photon laser scanning microscopy (TPLSM) combined with FITC-dextran vascular labeling to observe BBB leakage in living mice. The Sham group presented with a normal vascular structure whereas the Ischemia group displayed “sausage-like” deformations. QKL treatments reversed cerebrovascular structural changes after cerebral ischemia (Figure 5(e)). BBB leakage was observed under TPLSM in living mice (yellow arrows). FITC-dextran leakage was observed around the damaged microvascular tissue in the Ischemia group. The QKL treatment alleviated this leakage which means that QKL protects the BBB. To the best of our knowledge, this study is the first to use TPSLM to examine the effects of QKL on cerebral BBB dysfunction after tMCAO in living animals.

### 3.6. QKL Attenuates the Tight Junction Degradation Induced by Ischemic Stroke

**Figure 6:**
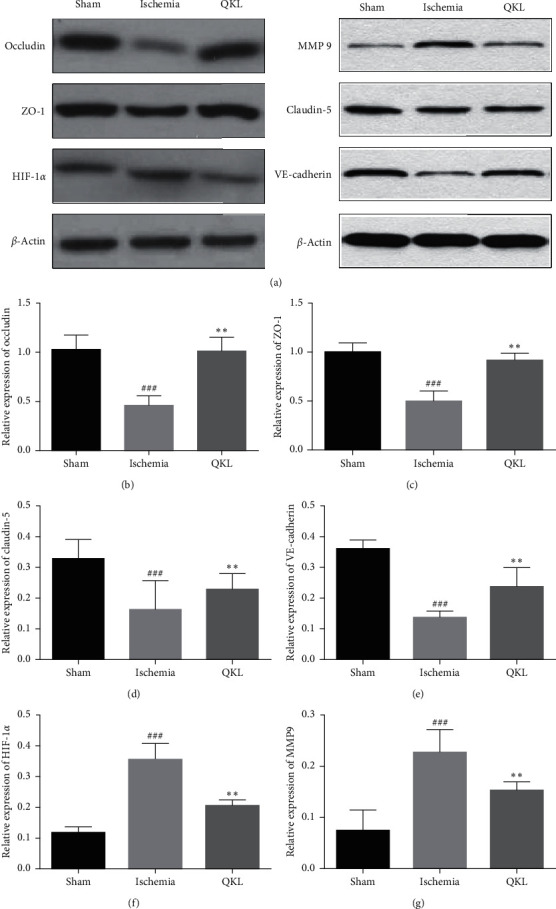
QKL protected tight junction degeneration and inhibited HIF-1*α* and MMP-9 activation. (a–e) Expression of occludin, ZO-1, claudin-5, and VE-cadherin was analyzed by western blotting and corresponding quantitative data. (a, f, g) Expression of HIF-1*α* and MMP-9 was analyzed by western blotting and corresponding quantitative data. Data are reported as means ± SEM. ^###^*P* < 0.001 vs. Sham group, ^*∗∗*^*P* < 0.01 vs. Ischemia group (*n* = 6 per group).

We used western blotting analysis to measure the expression levels of tight junctions (TJs). As shown in Figures 6(a)–6(e), ischemic stroke significantly downregulated TJs including ZO-1, claudin-5, VE-Cadherin, and occludin. Western blotting showed that QKL upregulated ZO-1, claudin-5, VE-Cadherin, and occludin, compared to the Ischemia group. Therefore, QKL treatment mitigated TJ damage caused by ischemia/reperfusion (I/R) injury.

### 3.7. QKL Downregulates HIF-1*α* and MMP-9 and Attenuates BBB Injury

**Figure 7:**
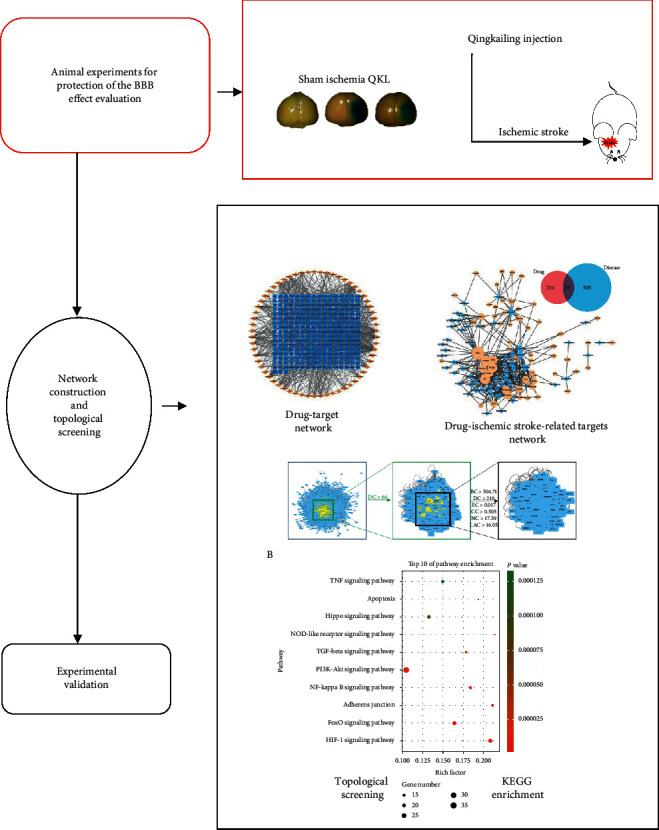
The image of the abbreviated article abstract.

We used western blotting analysis to measure the expression levels of HIF-1*α* and MMP-9. The expression level of HIF-1*α* in the Ischemia group was significantly higher than that in the Sham group. In contrast, QKL treatment downregulated HIF-1*α* (Figures 7(a) and 7(f)). Compared to the Ischemia group, QKL treatment markedly downregulated MMP-9 which was activated by ischemic stroke (Figures 7(a) and 7(g)). These results indicate that QKL mitigated cerebral BBB dysfunction by upregulating the tight junction proteins ZO-1, claudin-5, VE-Cadherin, and occludin and regulating HIF-1*α* and MMP-9 protein activation. HIF-1*α* is a nuclear transcription factor involved in ischemic injury. It is overactivated in response to ischemia/reperfusion injury and controls the expression of genes regulating the BBB.

## 4. Discussion

Qingkailing injection is a well-known SFDA-approved traditional Chinese medicine. It has been widely used in clinical practice for almost thirty years [46, 47]. We found that QKL decreased neurological deficits and infract volume formation in a tMCAO mice model. To evaluate biological mechanisms of QKL, network pharmacology was approached. In silico study indicated that targets of QKL participated in excitatory and inhibitory amino acid receptor and oxidative stress. Evidence supported the assumption that excitatory amino acids exert toxic effects during brain stroke [48]. Oxidative stress is a primary mediator of neurologic injury following ischemic stroke [49] and entails various mechanisms of the “ischemic cascade,” which leads to cell death and indicates that oxidative stress may become a potential therapeutic target of ischemic stroke. Cell junctions may play key roles in regulation of BBB stability and permeability to paracellular compounds [50]. It was thought that several QKL targets are expressed in cell junctions indicating a BBB modulating effect. QKL targets were also enriched in vasoconstriction progress. Therefore, QKL may also regulate cerebral blood perfusion and have multiple effects on the neurovascular system in response to ischemic stroke.

Previous studies reported the therapeutic effects of compounds identified in this study against various neurological disorders. Beta-sitosterol was reported to have antioxidative stress, antilipid peroxidation [51], anti-inflammatory [52], and neuroprotective [53] properties. It also inhibited NMDA receptor-mediated excitotoxicity related to ischemic stroke [54]. Stigmasterol was reported to participate in axon and dendrite development, the modulation of synaptic transmission [55], reduction of amyloidogenic amyloid precursor protein (APP) processing, inhibition of acetylcholine esterase, and amelioration of memory impairment [56]. Ursolic acid protected the ischemic brain in mice by activating Nrf2 pathway, inhibiting the TLR4/NF-*κ*B pathway, and regulating metalloprotease/anti-metalloprotease imbalance [57–59]. Pinoresinol [60] and tryptanthrin [61] induced a neuroinflammatory response and oxidative stress following brain ischemia. Unlike our results, baicalin, geniposide, cholic acid, and hyodeoxycholic acid were documented as the four important compounds of QKL [62]. Further studies should evaluate potential anti-ischemic stroke effect of these active compounds identified by our network pharmacological approach.

Previous studies have proposed estrogen deficiency as a risk factor for stroke in postmenopausal women [63]. Therefore, estrogen therapy could be protective here. Certain compounds in QKL were synergistic with the estrogen receptors ESR1 and ESR2. Activation of the estrogen receptors impedes BBB breakdown induced by ischemia/reperfusion injury and regulates tight junction protein levels in brain endothelial cells [64]. Phytoestrogens are, in fact, chemically, structurally, and functionally similar to estrogen [65]. Our results indicated 43 compounds which could potentially bind to estrogen receptors and accounted for 70 percent of all active compounds. In future research, the roles of phytoestrogens in neurovascular and cardiovascular diseases should be clarified using estrogen systems modulated by QKL.

Cerebral blood vessels blockage and BBB dysfunction are the main pathophysiological mechanisms of ischemic stroke. Protection of the BBB and correction of microcirculatory disturbances are two important therapeutic strategies in ischemic brain. HIF-1 is an important transcriptional factor of many genes associated with ischemic condition. It regulates angiogenesis, glucose metabolism, and cell survival during hypoxia and participates in apoptosis and inflammation [66]. Evidence indicated that HIF-1 signal pathway plays an important role in BBB disruption in ischemic stroke [67]. Our data suggested that the antistroke effect of QKL was strongly correlated with the HIF-1 signaling pathway and regulated BBB disruption via cell-cell junctions. Therefore, we designed an experiment to evaluate the BBB protective effect of QKL and its molecular mechanism related to the HIF-1 signaling pathway.

Commonly used methods for observing BBB dysfunction include Evans Blue detection and endogenous plasma IgG immunofluorescent staining. Two-photon imaging techniques enable the direct observation of brain tissue in living animals. We detected alterations in microvascular morphology induced by ischemic stroke. Early animal experiments showed that, in cerebral ischemia, astrocyte swelling compresses capillaries and pericytes regulate microvascular contraction and microcirculatory reflow [68]. Future studies should focus on the effects of QKL on glial cells. In addition to the glial cells reaction, increased BBB permeability narrows the lumen and lets plasma components deposit cellulose in response to tissue factors in the extravascular region [68]. Consequently, ischemic local microvessels may assume a “sausage-like” appearance, and their microperfusion is hindered [68, 69]. These phenomena are observed in the penumbra region in the early stages of tMCAO [48]. In future research, the relationships between the pericytes, astrocytes, and microvascular morphology should be investigated.

The BBB consists mainly of endothelial cells, their TJs, the basement membrane, pericytes, and perivascular glial cells. The TJs between the endothelial cells in the CNS vessels form an important structural basis for the BBB. Our results showed that QKL protected the TJs including ZO-1, claudin-5, VE-Cadherin, and occludin from damage caused by ischemic stroke. Upregulation of the TJs improved BBB function and reduced neurological deficit. Previous studies highlighted the roles of TJs in controlling inflammatory reactions [70]. Thus, further studies should focus on the cross-talk between BBB function and inflammatory response in order to elucidate the mechanisms of QKL against ischemic stroke.

Matrix metalloproteinases (MMPs) and others are closely associated with vascular endothelial growth factor (VEGF) and participate in acute BBB damage [71, 72].QKL was also reported to regulate matrix metalloproteinase-9 expression in animal models of cerebral I/R [73, 74], which is consistent with our results. In hypoxia/reperfusion, HIF-1*α* is synthesized and secreted into the cells in response to transcriptional activation of matrix metalloproteinase (MMP) in the astrocytes. HIF-1*α* also affects the structural integrity of the basement membrane and disrupts the BBB [75]. Degradation of the proteins and collagen associated with the BBB is also regulated by MMPs. Matrix metalloproteinases are zinc-dependent endopeptidases, and they degrade extracellular protein components [76]. MMP-9 has been extensively investigated [77] and was highly upregulated in an animal cerebral ischemia model and in human ischemic stroke. MMP-9 opens the BBB by breaking down TJs, increasing BBB permeability, and aggravating ischemic brain damage [78] (Figure 7).

## 5. Conclusions

A network pharmacology approach was used to reveal the biological mechanisms of QKL against ischemic stroke in the present study. A multiapproach, multitarget, and synergistic effect of bioactive compounds in QKL was visualized. Confirmed by experiments, QKL alleviated cerebral BBB dysfunction, regulated expression of TJs, and modulated HIF-1*α*/MMP-9 activation.

## Data Availability

The data used to support the findings of this study are available from the corresponding author upon request.

## Abbreviations

BBB: Blood-brain barrier
CCA: Common carotid artery
EB: Evans Blue
ECA: External carotid artery
EDA: Edaravone injection
ELISA: Enzyme-linked immunosorbent assay
FITC-dextran: Fluorescein isothiocyanate-labeled dextran
H&E: Hematoxylin and eosin staining
HIF-1: Hypoxia-inducible factor-1
ICA: Internal carotid artery
I/R: Ischemic reperfusion
MCAO: Middle cerebral artery occlusion
MMP-9: Matrix metalloprotein-9
Occludin: Tight junction protein occludin
PFA: Paraformaldehyde
QKL: Qingkailing injection
ROI: Region of interest
TEM: Transmission electron microscopy
TJ: Tight junction
TPLSM: Two-photon laser scanning microscopy
TTC: 2,3,5-Triphenyltetrazolium chloride
ZO-1: Zonula occludens-1.
